# Effects of olive oil and its fractions on oxidative stress and the liver's fatty acid composition in 2,4-Dichlorophenoxyacetic acid-treated rats

**DOI:** 10.1186/1743-7075-7-80

**Published:** 2010-10-29

**Authors:** Amel Nakbi, Wafa Tayeb, Abir Grissa, Manel Issaoui, Samia Dabbou, Issam Chargui, Meriem Ellouz, Abdelhedi Miled, Mohamed Hammami

**Affiliations:** 1 Laboratory of Biochemistry, UR03/ES08 'Human Nutrition & Metabolic Disorders', USCR Mass Spectrometry, Faculty of Medicine Monastir, Tunisia; 2 Laboratory of Histology and Cytogenetic, Faculty of Medicine Monastir, Tunisia; 3 Department of Biochemistry CHU F Hached, Sousse, Tunisia; 4 King Saud University, Riyadh, Saudi Arabia

## Abstract

**Background:**

Olive oil's beneficial effects are not only related to its high content of oleic acid, but also to the antioxidant potential of its polyphenols. In this study, we assess the effects of virgin olive oil and its fractions on 2,4-D- induced oxidative damage in the liver of rats.

**Methods:**

Male Wistar rats were randomly divided into eight groups of ten each: (C) a control group, (D) group that received 2,4-D (5 mg/kg b.w.), (D/EVOO) group treated with 2,4-D plus extra virgin olive oil, (D/OOHF) group that received 2,4-D plus hydrophilic fraction, (D/OOLF) group treated with 2,4-D plus lipophilic fraction, (EVOO) group that received only extra virgin olive oil, (OOHF) group given hydrophilic fraction and (OOLF) group treated with lipophilic fraction. These components were daily administered by gavage for 4 weeks.

**Results:**

A significant liver damage was observed in rats treated with 2,4-D via increased serum levels of transaminases and alkaline phosphatase, hepatic lipid peroxidation and decreased hepatic antioxidant enzyme activities, namely, superoxide dismutase, catalase, glutathione peroxidase, and glutathione reductase. The liver's fatty acid composition was also significantly modified with 2,4-D exposure. However, extra virgin olive oil and hydrophilic fraction intake during 2,4-D treatment induced a significant increase in the antioxidant enzyme activities and a decrease in the conjugated dienes (CD) and thiobarbituric acid-reactive substances (TBARs) levels in the liver. The lipophilic fraction supplemented to 2,4-D- treated rats did not show any improvement in the liver oxidative status while a marked improvement was detected in the hepatic fatty acid composition of rats supplemented with olive oil and the two fractions.

**Conclusion:**

We concluded that the protective effect of olive oil against oxidative damage induced by 2,4-D is mainly related to the antioxidant potential of its hydrophilic fraction.

## Background

Oxidative damage is a major contributor to the development of cardiovascular disease, cancer and neurodegenerative disorders. In healthy individuals, the generation of reactive oxygen species (ROS) is well balanced by the counterbalancing act of antioxidant defenses. Hence, an imbalance between ROS generation and antioxidant status in favor of the former has been described as oxidative stress [1]. ROS are constantly formed as by-products of normal metabolic reactions and their formation is accelerated by accidental exposure to occupational chemicals like pesticides.

Because of its relatively moderate toxicity, 2,4-Dichlorophenoxyacetic acid (2,4-D) has become one of the most widely used herbicides. Several reports have shown that 2,4-D produces oxidative stress and/or depletes antioxidants both *in vitro *and *in vivo*. *In vitro *studies have mainly investigated the effect of the herbicide on hepatocytes [2] and red blood cells [3,4]. While, *in vivo *oxidative activity has been shown in different species including plants [5], fish [6,7] and rats [8].

Olive oil is an integral ingredient in the Mediterranean diet. There is growing evidence that it may have great health benefits including the reduction in coronary heart disease risk, the prevention of some cancers and the modification of immune and inflammatory responses [9-11]. Virgin olive oil appears to be a functional food with various components such as monounsaturated fatty acids that may have nutritional benefits. It is also a good source of phytochemicals, including polyphenolic compounds [12,13]. It is known that an increased consumption of monounsaturated fatty acids (MUFA) instead of polyunsaturated fatty acids (PUFA) reduces the risk of atherosclerosis because it decreases the circulating lipoprotein's sensitivity to peroxidation [14].

Furthermore, the dietary MUFA healthy effects were attributed to decreased endothelial activation [15], and LDL susceptibility to oxidation [16]. In recent years, scientists have focused on the preventive effects of phenols against degenerative diseases mediated by the ROS. It has been reported that the phenolic compounds are able to interact with the biological systems and act as bioactive molecules. They are particularly important inhibitors of lipid peroxidation [17], and are believed to be effective through their free radical scavenging and metal-chelating properties [18,19]. In experimental studies, olive oil phenolic compounds showed strong antioxidant properties against lipids, DNA and LDL oxidation [20]. Hydroxytyrosol (2-(3,4 dihydroxyphenyl)ethanol, DPE), one of the phenolic compounds present in extra virgin olive oil, has been suggested to be a potent antioxidant, thus contributing to the beneficial properties of olive oil [21]. DPE administration has been shown to reduce the consequences of passive smoking-induced oxidative stress [22], prevent LDL oxidation [23] and platelet aggregation [24] and inhibit leukocyte 5-lipoxygenases [25]. DPE has shown efficacy in preventing oxidative stress in the liver of rats intoxicated by cadmium [26]. In addition, when human hepatoma HepG2 cells were pre-treated with DPE for 2 or 20 h prior to submission to tert-butylhydroperoxide-induced oxidative stress, cell toxicity was completely prevented, indicating that the antioxidant-treated cells were totally protected against the oxidative insult [27]. However, the liver is not only the main target for phenolic antioxidants once absorbed from the gastrointestinal tract but is the major place for phenolic metabolism. Therefore, studies dealing with the effect of antioxidant dietary phenolics on the liver should be given priority. The literature data on olive oil polyphenols is mainly concerned with purified compounds, while the antioxidant properties of the total fraction of the lipophilic or hydrophilic components have been poorly investigated. Being a complex mixture of compounds, the study of the protective effect could be more representative than of a single component.

The present study investigates the effect of dietary supplementation of olive oil, hydrophilic and lipophylic fractions on oxidative stress and liver fatty acid composition of 2,4-D- treated rats. In this context, we explored the hypothesis that, owing to its high content of natural antioxidants, olive oil could reduce 2,4-D-induced oxidative damage in rats.

## Materials and methods

### Materials

2, 4-D commercial formulation (Désormone Lourd) consists of 600 g/l 2,4-D Ester butylglycol with *H.96064 *register number. 2-Thiobarbituric acid (TBA) was obtained from Sigma Chemicals Co (Taufkirchen, Germany). 1, 1, 3, 3-tetramethoxypropane were purchased from Sigma Chemical Co. (St. Louis, MO). Folin-Ciocalteu phenol reagent was purchased from Fluka Biochemika (Buchs, Switzerland). All the other chemicals used were of analytical grade and were obtained from Sigma Chemicals Co or Merck (Darmstadt, Germany).

### Oil sample analysis

The used extra virgin olive oil (EVOO) was harvested from the North of Tunisia. The olive oil hydrophilic fraction (OOHF) was extracted from EVOO by the Montedoro method [28]. The olive oil lipophilic fraction (OOLF) was obtained from EVOO as follows: EVOO was homogenized for 1 min with water (1:1, v/v) and the oil was separated by centrifugation. This procedure was repeated six times. Then, the oil fraction (OOLF) was filtered through a cellulose acetate membrane. It can be seen that the phenolic compounds were efficiently removed from EVOO, being significantly reduced in the lipophilic fraction. The procedure employed to produce the OOLF, at variance with a washing process, has been developed to selectively eliminate hydrophilic substances, such as phenolic compounds, leaving unmodified the other olive oil components such as tocopherols and fatty acids. Different fractions were daily and freshly prepared and their composition was checked at the end of the treatment.

Fatty acids were converted into fatty acid methyl esters (FAMEs) prepared by dissolving 0.1 g of EVOO or OOLF in 2 ml of heptane and 0.2 ml of KOH (0.2 N) in methanol and incubated for 1 hour. Individual FAMES were separated and quantified by gas chromatography using model 5890 series II instrument (Hewlett-Packard Ca Palo Alto, Calif. USA) equipped with a flame ionisation detector and a fused silica capillary column HP - INNOWAX (30 m length × 0.25 mm i.d. and 0.25 μm of film thickness). The temperature was programmed to increase from 170 to 270°C at a rate of 5°C/min. Nitrogen ultra was used as carrier gas. The results were expressed as relative area percent of the total FAMES [29].

Oxidative stability was evaluated by the Rancimat apparatus (Model 743, Metrohm Schweiz AG, Zofingen, Switzerland) using 3 g of oil heated to 120°C with a 20 l/h air flow [30]. Stability was expressed as oxidation induction time (hours).

Carotenoids and chlorophylls (mg/kg oil) were determined at 470 and 670 nm, respectively, in cyclohexane using the specific extinction values according to Minguez Mosquera's method [31].

The phenolic compounds were extracted, estimated colorimetrically at 765 nm using the Folin-Ciocalteau reagent, and expressed as hydroxytyrosol equivalents as reported by Montedero et al. [28].

α-Tocopherol was evaluated according to Gimeno et al. [32] as follows: the sample was diluted with *n*-hexane (1:10), the mixture was vortexed and 200 μl was transferred to a test tube containing 600 μl of methanol and 200 μl of internal standard (300 μg/ml). HPLC separation was carried out on a Hewlett-Packard system (Waldbronn, Germany) equipped with a HP-1100 pump, a Rheodyne model 7725 injector (Cotati, CA, USA, loop volume 20 μl), a HP-1200 M multi-array detector and a Supelcosil ODS-2 column (150 × 4.5 mm id., film thickness 5 μm).

### Animal treatment

Male adult Wistar rats (Central Pharmacy, Tunisia), weighing about 200 to 230 g, were housed at 22 ± 3°C, with 12- hour light-dark periods, a 40% minimum relative humidity and free access to water and standard diet (SICO, Sfax Tunisia). After acclimatization to the laboratory conditions for one week, the animals were divided into 8 groups of 10 animals each. Group (C) included the control animals and received 1 ml of distilled water gavage daily and a standard diet. Group (D) was gavaged a daily dose of 2,4-D at a 5 mg/kg body weight concentration and fed with the standard diet. Group (D/EVOO) was treated simultaneously with 2,4-D at a dose of 5 mg/kg b.w. and EVOO (300 μl) daily by gavage. Groups (D/OOHF) and (D/OOLF) received daily 5 mg/kg b.w. of 2,4-D followed by hydrophilic fraction supplementation (1 ml of OOHF extracted from 5 g of EVOO) and lipophilic faction (300 μl) by gavage, respectively. The animals in control groups (EVOO), (OOHF), and (OOLF) were given EVOO (300 μl), hydrophilic fraction (1 ml) and lipophilic fraction (300 μl), respectively. The animals supplemented EVOO and OOHF received approximately the same amount of phenols: 0.17 mg and 0.19 mg/day, respectively.

Each group was kept on treatment for 4 weeks. Water and food consumption and the individual animal body-weight were recorded daily throughout the experiment. At the end of the experimental period, the rats were kept fasting overnight and were sacrificed under diethyl ether anaesthesia.

All the breeding phases and all experiments were carried out in compliance with the rules of the Tunisian Society for the Care and Use of Laboratory Animals. All experiments were conducted at the animal facilities of the faculty of Medicine, Monastir; with the approval of the Faculty of Medicine Ethics committee.

### Blood and tissue collections

Blood was drawn by cardiac puncture and the livers were collected for biochemical examinations. Serum was obtained by centrifugation at 3000 g for 10 min and stored at -80°C in aliquots until the analysis. The livers were washed with ice-cold physiological saline solution (0.9%), blotted dry and weighed. The tissues were homogenized for 30 seconds in 10 volumes of ice-cold 10 mmol/l phosphate-buffered saline (pH 7.4) containing 1.15% KCl. The homogenate was subjected to a 6000 g centrifugation at 4°C for 15 min. The supernatant fractions were collected and stored at -80°C until analysis. The protein content of supernatant fractions was determined according to Bradford [33].

### Biochemical analysis of liver functions

Enzyme activities of aspartate aminotransferase (AST), alanine aminotransferase (ALT), alkaline phosphatase (ALP), γ-glutamyltransferase (γGT), and total bilirubin in serum were measured using the commercially available diagnostic kits supplied by Randox Laboratories (Ardmore, Northern Ireland, UK).

### Liver lipoperoxidation

The level of lipid peroxidation products was measured as thiobarbituric acid reactive metabolites (TBA-rm) according to Yagi [34]. 125 μl of serum or supernatants were homogenized by sonication with 50 μl of TBS, 125 μl of TCA-BHT in order to precipitate proteins and then centrifuged (1000 g, 10 min, 4°C). 200 μl of supernatant were mixed with 40 μl of HCl (0.6 M) and 160 μl of TBA dissolved in Tris and the mixture was heated at 80°C for 10 min. The absorbance of the resultant supernatant was read at 530 nm. The TBA-rm amount was calculated using a 156 mM-^1 ^cm-^1 ^extinction coefficient.

Another indicator of the lipid peroxidation is the conjugated diene (CD) which is measured as described by Esterbauer et al. [35]. The results were expressed as μmol hydroperoxides/mg protein using ε = 2.52 × 10^4 ^M^-1 ^cm^-1^.

### Liver activities of antioxidant enzymes

The antioxidant enzyme activities were analysed using a BioRad UV-Visible spectrophotometer with a "kinetics" program (BioRad, Mares la Coquette, France). The measurement of superoxide dismutase (SOD), glutathione peroxidase (GSH-Px) and Glutathione reductase (GR) activities in supernatants were performed by the commercially available diagnostic kits supplied by Randox Laboratories. The Catalase (CAT) activity was measured at 25°C according to Aebi's method [36] by calculating H_2_O_2 _concentration decrease at 240 nm.

### Liver fatty acid composition

Fatty acids were analysed as fatty acid methyl esters (FAMEs) by gas chromatography analysis as described by Giacometti et al. [37]. Briefly, the total lipids were extracted from the tissue homogenates by the modified method mentioned by Folch et al. [38] using a chloroform-methanol (2:1, v/v) solvent system containing 0.01% butylated hydroxytoluene as an antioxidant. The aliquots of the total lipids were converted to methyl esters with a mixture of methanol-hexane-H_2_SO_4 _(75:25:1, v/v/v) as the methylation reagent at 90°C for 90 min. FAMEs were analyzed in duplicate, and 1 μl of each sample was injected into the gas chromatography system (Hewlett Packard, Palo Alto, Calif.) equipped with a flame ionization detector and a polar fused silica capillary column HP-Innowax with cross-linked PEG, Carbowax 20 M (30 m × 0.25 mm id. and 0.25 μm as film thickness). The oven temperature was programmed to increase from 180°C to 250°C at a rate of 10°C/min. The injector and detector temperatures were 220°C and 280°C, respectively. FAMEs were identified by comparing their retention times with those of individual standards.

### Statistical analysis

The data were analyzed using the Statistical Package for Social Sciences (SPSS) programme, release 11.0 for Windows (SPSS, Chicago, IL, USA). In each assay, the experimental data represent the mean of ten independent assays ± standard deviations. The results were analyzed using the *Student t *test for comparison between the different treatment groups. Tukey's test was used to determine any significant differences between group means of liver fatty acid composition (one-way ANOVA test). The statistical significance was set at *p *< 0.05.

## Results

### Analytical parameters of extra virgin olive oil, its fractions and standard diet

The used olive oil's analytical parameters (fatty acids, oxidative stability and antioxidant composition) are shown in Table 1. Extra virgin olive oil contained 17.3% saturates (palmitic and stearic acids), 66.2% monounsaturates (mainly oleic acid) and 15% polyunsaturates, whereas the standard diet consisted of 13% saturates (palmitic and stearic acids), 30% monounsaturates and 54.5% polyunsaturates (high content of linoleic acid). Some significant differences were noted in the amount of phenols of the oil tested. In fact, EVOO and OOHF contained high amounts of phenols (579.2 and 192 mg/kg, respectively) while OOLF was deprived from phenols and presented the same amount of α-tocopherol as the EVOO (Table 1).

**Table 1:** Mean values of analytical parameters, fatty acids composition (%), oxidative stability and antioxidant content of extra virgin olive oil, hydrophilic fraction, lipophilic fractions and standard diet fed to rat.

	Extra virgin olive oil (EVOO)	Lipophilic fraction (OOLF)	Hydrophilic fraction (OOHF)	Standard diet
Palmitic acid [%]	10.28 ± 0.04^a^	10.40 ± 0.01^a^	-	11.62 ± 0.85^a^
Palmitoleic acid	0.77 ± 0.30^a^	0.79 ± 0.60^a^	-	0.10 ± 0.04 ^a^
Stearic acid	3.39 ± 0.14^a^	3.56 ± 0.37^a^	-	1.15 ± 0.32 ^b^
Oleic acid	64.80 ± 1.99 ^a^	62.58 ± 3.71 ^a^	-	29.73 ± 1.12 ^b^
Linoleic acid	14.34 ± 0.90 ^b^	15.04 ± 0.54 ^b^	-	53.89 ± 1.49^a^
Linolenic acid	0.64 ± 0.04^a^	0.68 ± 0.03 ^a^	-	0.61 ± 0.02 ^a^
Arachidic acid	0.74 ± 0.05^a^	0.78 ± 0.02^a^	-	0.09 ± 0.04 ^b^
Gadoleic acid	0.62 ± 0.03^a^	0.57 ± 0.03^a^	-	0.14 ± 0.04 ^b^
Behenic acid	2.84 ± 0.37^a^	2.87 ± 0.80^a^	-	0.22 ± 0.10 ^b^
SFA	17.28 ± 0.22^a^	17.62 ± 0.42 ^a^	-	13.08 ± 0.21 ^b^
MUFA	66.20 ± 2.34 ^a^	63.95 ± 3.07 ^a^	-	29.97 ± 1.32 ^b^
PUFA	14.99 ± 0.94^b^	15.72 ± 0.57^b^	-	54.50 ± 2.12^a^
MUFA/PUFA	4.43 ± 0.43 ^a^	4.07 ± 0.04^a^	-	0.54 ± 0.32 ^b^

OSI (h)	9.6^a^	4.7 ^b^	-	-
Chlorophylls (mg/kg)	12.65 ± 0.29^a^	5.4 ± 0.76 ^b^	-	ND
β-Carotene (mg/kg)	7.15 ± 0.20 ^a^	5.43 ± 0.08 ^b^	-	ND
Total polyphenols (mg/kg)	579.17 ± 71.40*^a^	-	192.00 ± 11.59**^b^	ND
α- tocopherol (mg/kg)	484.56 ± 11.11^a^	491.64 ± 10.36^a^	-	224 ± 11.86 ^b^

Data are expressed as mean values ± of three independent experiments. SFA: saturated fatty acids; MUFA: monounsaturated fatty acid; PUFA: polyunsaturated fatty acid; OSI: oxidative stability Index. ND: indicates not determined

Values followed by same letters are not significantly different. (Tukey's test, *p *< 0.05)

* As hydroxytyrosol equivalents (mg/kg EVOO)

** As expressed mg of hydroxytyrosol equivalents/kg of OOHF

### Biochemical indicator of liver functions

All the results from various treatment groups were compared with their normal controls (C). However, results from 2,4-D + extra virgin olive oil (D/EVOO), 2,4-D + olive oil hydrophilic fraction (D/OOHF) or 2,4-D + olive oil lipophilic fraction (D/OOLF) groups were also compared with the data of 2,4-D-treated group (D). During this study, death was not observed during the experimental period. The rats exhibited a normal behavior in comparison to the control group. There were no significant differences between the treated and control rats in body weight gain during the experiment (Table 2). However, the relative liver weight was significantly increased in 2,4-D treated rats supplemented or not with olive oil and hydrophilic fraction compared to controls.

**Table 2:** Body weight, weight gain, relative liver weight of control and experimental rats

Parameters and groups	Initial body weight (g)	Final body weight (g)	Weight gain (%)	Relative liver weight (g/100 g body weight)
C	216.80 ± 18.10	273.70 ± 27.10	26.16 ± 4.05	3.11 ± 0.32
D	220.00 ± 11.11	276.70 ± 15.23	25.62 ± 5.66	3.56 ± 0.30^§§^
D/EVOO	222.80 ± 19.03	280.60 ± 19.77	26.21 ± 6.15	3.59 ± 0.23^§§^
D/OOHF	222.60 ± 25.29	279.10 ± 34.74	25.32 ± 5.67	3.47 ± 0.28^§^
D/OOLF	219.10 ± 16.17	277.40 ± 21.66	26.69 ± 5.90	3.36 ± 0.25
EVOO	221.30 ± 22.84	271.10 ± 22.96	22.81 ± 6.10	3.50 ± 0.21^§^
OOHF	225.50 ± 23.10	286.10 ± 25.86	27.08 ± 4.33	3.43 ± 0.21^§^
OOLF	221.50 ± 30.64	284.50 ± 29.53	29.82 ± 6.85	3.36 ± 0.11

Data are expressed as means ± SD (n = 10 rats per group). C: controls group, D: 2, 4-D treated group, D/EVOO: 2, 4-D plus extra virgin olive oil, D/OOHF: 2, 4-D plus hydrophilic fraction, D/OOLF: 2, 4-D plus lipophilic fraction, EVOO: extra virgin olive oil treated group alone, OOHF: group treated with hydrophilic fraction of olive oil alone, OOLF: group treated with lipophilic fraction of olive oil alone. Comparison between groups was made using unpaired Student t test.

* p < .05 (compared to Group D)

§§ p < .01; § p < .05 (compared to controls C)

The extent of liver damage sustained following exposure to 2,4-D is shown in Table 3. The serum levels of AST, ALT, ALP, γ-GT and total Bilirubin were significantly higher in 2,4-D-treated rats (D) than in the control group (C). The increase of AST, ALT activities and total bilirubin was markedly reduced in the presence of olive oil and its fractions (*p *< 0.05) while bringing back their rates towards the normal level found in the control group although ALP and γ-GT activities did not alter significantly following olive oil and its extracts administered to 2,4-D-treated rats. So, a significant increase of ALP and γ-GT activities was noted in animals treated with lipophilic extract and 2,4-D compared to the controls. In the control animals treated with olive oil or the two extracts alone no significant changes were observed except for γ-GT activity which significantly increased in animals treated with lipophilic extract alone compared to controls.

**Table 3:** Effects of extra virgin olive oil (EVOO) and its fractions (OOHF and OOLF) on biochemical indicators of liver function (AST, ALT, ALP, γ-GT and total bilirubin) in serum of rat treated or not with 2,4-D.

Groups	AST (U/L)	ALT (U/L)	ALP (U/L)	γ-GT (U/L)	T-Bilirubin (U/I)
C	96.40 ± 17.07	33.75 ± 3.24	109.80 ± 13.62	20.20 ± 3.76	5.66 ± 1.18
D	138.16 ± 12.44^§§^	57.50 ± 10.60^§§^	157.57 ± 27.09^§§^	32.00 ± 2.94^§§^	12.05 ± 0.81^§§^
D/EVOO	97.71 ± 13.57‍ **	38.00 ± 3.16**	123.33 ± 20.81	34.14 ± 5.81^§§^	7.00 ± 0.91**
D/OOHF	94.16 ± 10.72**	35.40 ± 3.20**	127 ± 23.64	30.14 ± 12.40	7.56 ± 1.70**^§^
D/OOLF	103.50 ± 12.55**	31.28 ± 4.46**	194.25 ± 12.81**^§§^	34.60 ± 12.09^§^	7.42 ± 0.61**^§^
EVOO	94.60 ± 6.69	34.83 ± 4.35	107.00 ± 24.73	25.00 ± 8.64	6.14 ± 1.09
OOHF	95.83 ± 10.79	29.44 ± 3.43	123.60 ± 28.83	22.57 ± 7.36	6.86 ± 1.33
OOLF	96.37 ± 9.85	32.66 ± 3.44	119.00 ± 10.98	30.60 ± 7.16^§^	5.58 ± 1.68

Student t test (C: controls group, D: 2,4-D treated group, D/EVOO: 2,4-D plus extra virgin olive oil, D/OOHF: 2,4-D plus hydrophilic fraction, D/OOLF: 2,4-D plus lipophilic fraction, EVOO: extra virgin olive oil treated group alone, OOHF: group treated with hydrophilic fraction of olive oil, OOLF: group treated with lipophilic fraction of olive oil.

** p <.01; * p < .05 (compared to Group D)

^§§ ^p < .01; ^§ ^p < .05 (compared to controls C)

### Activities of liver antioxidant enzymes

The rat's exposure to 2,4-D resulted in liver injury and extensive oxidative damage as manifested by the significant decline in SOD, CAT, GPx and GR enzymes levels by -33%, -39%, -60% and -26%, respectively, compared to controls (Table 4). In contrast, treatment with olive oil or hydrophilic extract in association with 2,4-D increased the levels of antioxidant enzymes, showing similar activities to the control group. However, lipophilic fraction treatment plus 2,4-D restored the levels of the oxidative damage induced by 2,4-D, indicating that the components of this extract did not influence the potential of 2,4-D as an oxidative stress inducer in the liver. When compared to the control group, no statistically significant differences were detected in the groups which were administered olive oil or hydrophilic extract alone with respect to liver parameter levels. However, the oral administration of lipophilic extract alone caused a significant decrease of SOD, CAT and GR activities in the rats' livers.

**Table 4:** Effects of extra virgin olive oil (EVOO) and its fractions (OOHF and OOLF) on antioxidant enzymes activities in liver of rat treated or not with 2,4-D.

Groups	Antioxidant enzyme activity (U/mg liver protein)			
	
	SOD	CAT	GPX	GR
C	9.49 ± 1.34	23.42 ± 0.53	0.75 ± 0.20	0.05 ± 0.0035
D	6.71 ± 1.58^§^	14.51 ± 1.01^§§^	0.30 ± 0.09^§^	0.037 ± 0.007^§^
D/EVOO	13.38 ± 1.76*	20.29 ± 0.68^§§^**	0.80 ± 0.27*	0.056 ± 0.01*
D/OOHF	8.13 ± 0.97	22.50 ± 1.29**	0.76 ± 0.27*	0.053 ± 0.005*
D/OOLF	6.45 ± 0.96^§^	15.96 ± 0.28^§§^	0.55 ± 0.05*	0.031 ± 0.011^§^
EVOO	9.65 ± 1.13	22.50 ± 1.29	0.86 ± 0.39	0.051 ± 0.003
OOHF	9.53 ± 0.59	22.71 ± 0.52	0.74 ± 0.17	0.052 ± 0.008
OOLF	6.47 ± 1.33^§^	18.59 ± 1.09^§§^	0.51 ± 0.16	0.034 ± 0.013^§^

Data are expressed as means ± SD (n = 10 rats per group). Comparison between groups was made using unpaired Student t test. (C: controls group, D: 2, 4-D treated group, D/EVOO: 2, 4-D + extra virgin olive oil, D/OOHF: 2, 4-D+ hydrophilic fraction, D/OOLF: 2,4-D+ lipophilic fraction, EVOO: extra virgin olive oil treated group alone, OOHF: group treated with hydrophilic fraction of olive oil, OOLF: group treated with lipophilic fraction of olive oil.* P < .05 vs 2,4-D group; ** P < .01 vs 2,4-D group. ^§ ^P < .05 vs. control group; ^§§ ^P < .01 vs. control group.

### The liver's lipid peroxidation

**Figure 1:**
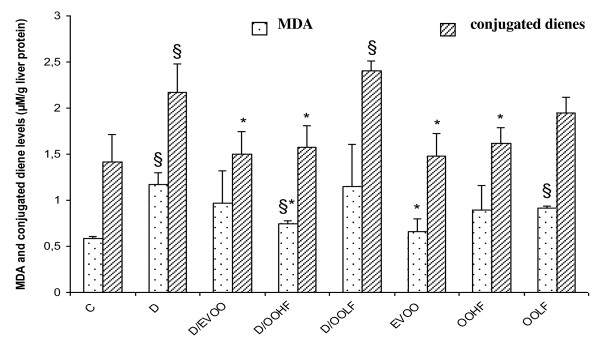
**Malondialdehyde (MDA) and conjugated dienes (CD) levels in liver of rat treated or not with 2,4-D under effects of supplemented extra virgin olive oil (EVOO) and its fractions (OOHF and OOLF)**. C: controls group, D: 2, 4-D treated group, D/EVOO: 2, 4-D + extra virgin olive oil, D/OOHF: 2, 4-D+ hydrophilic fraction, D/OOLF: 2,4-D+ lipophilic fraction, EVOO: extra virgin olive oil treated group alone, OOHF: group treated with hydrophilic fraction of olive oil, OOLF: group treated with lipophilic fraction of olive oil. Data are expressed as means ± SD (n = 10 rats per group). Comparison between groups was made using unpaired *Student t *test. * *p *< .05 vs 2,4-D group; § *p *< .05 vs control group.

The influence of sub acute exposure to 2,4-D and the administration of olive oil and its two extracts on lipid peroxidation in the liver is shown in Figure 1. In fact, 2,4-D at 5 mg/kg/day significantly increased the hepatic lipid peroxidation as indicated by the higher amounts of malondialdehyde (MDA) formed and conjugated diene (CD). However, the administration of olive oil (300 μl/day) and hydrophilic extract (1 ml) in association with 2,4-D reduced MDA hepatic levels to 82 and 64%, respectively, and CD to 69 and 72%, respectively, compared to the 2,4-D group (Figure 1). On the other hand, the administration of lipophilic fraction to 2,4-D-treated animals showed no significant differences in MDA and CD levels compared to the 2,4-D-treated group. These findings indicated that the free radicals released in the liver were effectively scavenged when treated with olive oil hydrophilic extract.

### The liver's fatty acid composition

Fatty acid composition of the liver homogenate is shown in Table 5. The major fatty acids in the livers of all groups were palmitic (16:0), stearic (18:0), oleic (18:1n-9), linoleic (18:2n-6) and arachidonic (20:4n-6) acids. The slight increase in the saturated fatty acid (SFA) content (such as myristic, stearic and arachidic acids) and the decreased level of MUFA (such as myristoleic, oleic and nervonic acids) and PUFA (such as α-linolenic, eicosapentaenoic and docosapentaenoic acids) characterized the liver's fatty acid composition of pesticide-treated rats compared to controls. The hepatic fatty acid in animals administered olive oil or its extracts in combination with 2,4-D had significantly lower total SFA levels and higher total MUFA proportions than 2,4-D treated rats (*p *< 0.01). This effect was mainly due to an increase in C14:1, C18:1 and C24:1 at the extent of C14:0, C18:0 and C20:0. PUFA proportion was somewhat lowered in the liver of animals supplemented with olive oil and treated or not with 2,4-D. This was caused by a reduction of C18:2, C20:4 and C20:2 (Table 5). Therefore, the ratio of MUFA to PUFA increased significantly in the liver of animals in these groups compared to control animals (from 0.43 to 0.53 and 0.63, respectively, (*p *< 0.01)). The total SFA, MUFA and PUFA levels in the livers of rats' supplemented hydrophilic fraction of olive oil remained unchanged compared to control animals due to dietary antioxidant compound supply.

**Table 5:** Effects of extra virgin olive oil (EVOO) and its fractions (OOHF and OOLF) on liver fatty acid composition of rat treated or not with 2,4-D.

Fatty acid (%)	C	D	D/EVOO	D/OOHF	D/OOLF	EVOO	OOHF	OOLF
C14:0	1.15 ± 0.11^ab^	1.35 ± 0.11 ^a^	1.18 ± 016 ^ab^	1.19 ± 0.15 ^ab^	1.26 ± 0.15 ^ab^	1.05 ± 0.15 ^b^	1.13 ± 0.18 ^ab^	1.16 ± 0.16 ^ab^
C14:1	0.46 ± 0.05 ^a^	0.31 ± 0.06 ^b^	0.48 ± 0.07 ^a^	0.47 ± 0.06 ^a^	0.44 ± 0.09 ^a^	0.48 ± 0.06 ^a^	0.48 ± 0.06 ^a^	0.40 ± 0.05 ^ab^
C16:0	17.59 ± 0.81 ^a^	17.27 ± 0.72 ^ab^	16.71 ± 0.39 ^ab^	17.36 ± 0.38 ^ab^	16.64 ± 0.75 ^ab^	16.38 ± 0.22 ^b^	16.64 ± 0.73 ^ab^	16.98 ± 0.78 ^ab^
C16:1	1.46 ± 0.25 ^a^	1.26 ± 0.11 ^a^	1.45 ± 0.31 ^a^	1.50 ± 0.32 ^a^	1.42 ± 0.26 ^a^	1.49 ± 0.28 ^a^	1.39 ± 0.07 ^a^	1.48 ± 0.26 ^a^
C18:0	19.24 ± 0.54 ^c^	24.80 ± 0.71 ^a^	20.66 ± 0.46 ^b^	21.68 ± 0.49 ^b^	22.47 ± 1.86 ^b^	19.46 ± 0.26 ^c^	19.01 ± 0.44 ^c^	19.61 ± 0.90 ^c^
C18:1	14.77 ± 0.42 ^b^	11.87 ± 0.30 ^c^	16.90 ± 0.70 ^b^	14.65 ± 0.20 ^b^	16.03 ± 0.50 ^b^	19.17 ± 0.72 ^a^	15.36 ± 0.77 ^b^	15.92 ± 0.37 ^b^
C18:2	13.74 ± 0.68 ^ab^	14.17 ± 0.56 ^a^	12.70 ± 0.70 ^bc^	13.15 ± 0.82 ^abc^	14.27 ± 0.87 ^a^	12.12 ± 0.86 ^c^	13.04 ± 0.63 ^abc^	14.10 ± 0.61 ^a^
C18:3	0.47 ± 0.06 ^a^	0.18 ± 0.06 ^c^	0.28 ± 0.08 ^bc^	0.29 ± 0.03 ^bc^	0.30 ± 0.04 ^b^	0.46 ± 0.07 ^a^	0.49 ± 0.08 ^a^	0.51 ± 0.09 ^a^
C20:0	0.35 ± 0.02 ^c^	0.52 ± 0.18 ^a^	0.40 ± 0.04 ^bc^	0.39 ± 0.06 ^bc^	0.47 ± 0.07 ^ab^	0.33 ± 0.06 ^c^	0.37 ± 0.05 ^bc^	0.37 ± 0.07 ^bc^
C20:1	0.50 ± 0.04 ^ab^	0.37 ± 0.04 ^b^	0.49 ± 0.12 ^ab^	0.54 ± 0.12 ^a^	0.48 ± 0.05 ^ab^	0.59 ± 0.13 ^a^	0.55 ± 0.12 ^a^	0.59 ± 0.04 ^a^
C20:2	0.51 ± 0.07 ^ab^	0.64 ± 0.18 ^a^	0.44 ± 0.08 ^bc^	0.47 ± 0.10 ^abc^	0.48 ± 0.15 ^abc^	0.31 ± 0.09 ^c^	0.45 ± 0.10 ^bc^	0.40 ± 0.13 ^bc^
C20:3	0.74 ± 0.07 ^a^	0.47 ± 0.08 ^a^	0.65 ± 0.13 ^a^	0.66 ± 0.09 ^a^	0.40 ± 0.07 ^b^	0.62 ± 0.12 ^a^	0.65 ± 0.02 ^a^	0.36 ± 0.03 ^b^
C20:4	19.65 ± 1.29 ^ab^	17.77 ± 0.32 ^ab^	18.69 ± 1.73 ^ab^	19.26 ± 2.39 ^ab^	18.08 ± 2.01 ^ab^	16.96 ± 2.61 ^b^	20.80 ± 1.52 ^a^	18.33 ± 2.10 ^ab^
C20:5 EPA	0.51 ± 0.07 ^a^	0.36 ± 0.06 ^b^	0.48 ± 0.08 ^ab^	0.50 ± 0.06 ^ab^	0.47 ± 0.09 ^ab^	0.47 ± 0.06 ^ab^	0.53 ± 0.05 ^a^	0.51 ± 0.13 ^a^
C22:0	0.50 ± 0.11 ^a^	0.52 ± 0.08 ^a^	0.53 ± 0.13 ^a^	0.55 ± 0.11 ^a^	0.45 ± 0.12 ^a^	0.44 ± 0.10 ^a^	0.44 ± 0.10 ^a^	0.46 ± 0.11 ^a^
C22:4	0.32 ± 0.05 ^ab^	0.44 ± 0.18 ^a^	0.38 ± 0.08 ^ab^	0.36 ± 0.08 ^ab^	0.35 ± 0.10 ^ab^	0.28 ± 0.06 ^b^	0.37 ± 0.03 ^ab^	0.38 ± 0.15 ^ab^
C22:1	0.60 ± 0.07 ^a^	0.54 ± 0.08 ^a^	0.73 ± 0.15 ^a^	0.65 ± 0.12 ^a^	0.61 ± 0.17 ^a^	0.76 ± 0.20 ^a^	0.62 ± 0.15 ^a^	0.72 ± 0.04 ^a^
C22:5	0.65 ± 0.07 ^a^	0.51 ± 0.04 ^b^	0.74 ± 0.07 ^a^	0.62 ± 0.06 ^ab^	0.64 ± 0.10 ^a^	0.70 ± 0.05 ^a^	0.68 ± 0.09 ^a^	0.67 ± 0.06 ^a^
C24:0	0.45 ± 0.09 ^a^	0.47 ± 0.03 ^a^	0.37 ± 0.04 ^a^	0.41 ± 0.08 ^a^	0.40 ± 0.08 ^a^	0.38 ± 0.06 ^a^	0.42 ± 0.08 ^a^	0.40 ± 0.09 ^a^
C22:6 DHA	4.59 ± 0.39 ^a^	4.38 ± 0.35 ^a^	4.36 ± 0.28 ^a^	4.60 ± 0.31 ^a^	4.40 ± 0.31 ^a^	4.24 ± 0.32 ^a^	4.48 ± 0.26 ^a^	4.38 ± 0.22 ^a^
C24:1	0.32 ± 0.06 ^a^	0.19 ± 0.07 ^b^	0.38 ± 0.03 ^a^	0.33 ± 0.07 ^a^	0.34 ± 0.07 ^a^	0.40 ± 0.06 ^a^	0.37 ± 0.08 ^a^	0.38 ± 0.06 ^a^

ΣSFA	39.31 ± 0.97 ^cd^	44.95 ± 1.09 ^a^	39.88 ± 0.71 ^cd^	41.61 ± 0.80 ^b^	41.71 ± 2.30 ^b^	38.06 ± 0.25 ^d^	38.04 ± 1.07^d^	39.00 ± 1.47 ^cd^
ΣMUFA	18.12 ± 0.52 ^d^	14.56 ± 0.30 ^e^	20.47 ± 1.11 ^b^	18.17 ± 0.51 ^d^	19.34 ± 0.62 ^bc^	22.91 ± 0.80 ^a^	18.78 ± 0.70 ^cd^	19.52 ± 0.58 ^bc^
ΣPUFA	41.22 ± 1.39 ^a^	38.95 ± 0.79 ^b^	38.75 ± 1.98 ^b^	39.96 ± 2.17 ^a^	39.43 ± 2.75 ^a^	36.19 ± 1.32 ^b^	41.53 ± 1.32 ^a^	39.68 ± 1.95 ^a^
ΣUFA	59.34 ± 1.86 ^a^	53.51 ± 0.96 ^b^	59.23 ± 2.00 ^a^	58.14 ± 2.44 ^a^	58.77 ± 2.32 ^a^	59.10 ± 1.63 ^a^	60.32 ± 1.53 ^a^	59.20 ± 1.75 ^a^
MUFA/PUFA ratio	0.43 ± 0.007 ^c^	0.37 ± 0.008 ^d^	0.52 ± 0.04 ^b^	0.45 ± 0.02^c^	0.49 ± 0.04 ^bc^	0.63 ± 0.05 ^a^	0.45 ± 0.02 ^c^	0.49 ± 0.03 ^bc^
PUFA/SFA ratio	1.04 ± 0.04 ^ab^	0.86 ± 0.03^d^	0.97 ± 0.05^bc^	0.96 ± 0.04 ^bc^	0.94 ± 0.08 ^cd^	0.95 ± 0.04 ^cd^	1.09 ± 0.06 ^a^	1.01 ± 0.04 ^abc^
SFA/UFA ratio	0.66 ± 0.02 ^cd^	0.84 ± 0.02 ^a^	0.67 ± 0.03 ^cd^	0.71 ± 0.02 ^b^	0.71 ± 0.05^b^	0.64 ± 0.01 ^d^	0.63 ± 0.03 ^d^	0.69 ± 0.07 ^b c^

Data are expressed by mean values ± of three independent experiments. SFA: saturated fatty acids; MUFA: monounsaturated fatty acid; PUFA: polyunsaturated fatty acid. Comparison between groups was made using one-way analysis of variance (ANOVA) followed by the Tukey test. Values followed by same letters are not significantly different. (C: controls group, D: 2, 4-D treated group, D/EVOO: 2, 4-D + extra virgin olive oil, D/OOHF: 2, 4-D+ hydrophilic fraction, D/OOLF: 2,4-D+ lipophilic fraction, EVOO: extra virgin olive oil treated group alone, OOHF: group treated with hydrophilic fraction of olive oil, OOLF: group treated with lipophilic fraction of olive oil.

## Discussion

The increasing popularity of olive oil is mainly attributed to its antioxidant and anti-inflammatory effects which may help prevent disease in humans [39,40]. In the current study, an attempt has been made to assess the hepatoprotective potential of olive oil and the supplementation of its two fractions (hydrophilic and lipophilic) in animals subjected to 2,4-D intoxication. 2,4-D was preferred because of its wide use as a selective herbicide in the North of Tunisia for cereal culture. However, as a toxicological agent like other pesticides, it is conceivable that 2,4-D might interact primarily with the liver resulting in structural damage and changes in enzyme leakage and in the metabolism of the constituents. Some previous studies have looked at the *in vitro *effects of 2,4-D on the generation of oxidative stress, either at the mitochondrial level in hepatocytes or in red blood cells [2,4]. Furthermore, it has been reported that acute exposure to 2,4-D pesticides may induce oxidative stress in rats [8]. The authors found that the administration of 1.5 and 3 mg/d of 2,4-D for 25 days might affect antioxidant potential enzymes, the activity of hepatic damage enzymes and lipid peroxidation. Recently, other studies have investigated the effect of 2,4-D at 3 mg/kg b.w. for 4 weeks in jerboa (Jaculus orientalis), a wild animal of the sub desert highlands [41]. In fact, they showed that 2,4-D induces toxicity which affects energy metabolism, morphological disorders and oxidative stress. These results are in agreement with ours in that the treatment of 2,4-D at 5 mg/kg b.w. increased the marker enzymes activities in serum, the production of lipid peroxides and affected antioxidant defense in the liver of rats in comparison to controls. In fact, the enhanced activities of transaminases (AST and ALT), ALP and γ-GT, and the increased level of total biluribin revealed hepatic damage in the 2,4-D-treated group.

The antioxidant enzymes (SOD, GPx, GR and CAT) limit the effects of oxidant molecules on tissues and are active in the defense against oxidative cell injury thanks to the fact that they are free radical scavengers [42]. Consequently, in the current study, it can suggested that the significant decrease of the antioxidant enzyme activities and the increase of MDA and CD contents in the liver proved the failure of antioxidant defense system to overcome the influx of ROS generated by 2,4-D exposure. However, the oral administration of EVOO and the two extracts to 2,4-D-treated rats caused a modulation in the activity of the above enzymes and lipid peroxidation, which may have resulted from the stabilization of plasma membrane as well as the repair of the hepatic tissue damage caused by 2,4-D. This is supported by the view that serum levels of transaminases return to normal with the healing of hepatic parenchyma and the regeneration of hepatocytes [43]. In addition, depletion of the elevated bilirubin level together with the suppression of ALP activity in the serum of rats treated with EVOO and the two extracts suggests the biliary dysfunction of the rat's liver during sub acute injury with 2,4-D has been stabilized. This result was more pronounced in rats treated with EVOO, which reflects the synergic effect of the two fractions in restoring ALP and bilirubin serum levels. Furthermore, the oral administration of EVOO or the two extracts with 2,4-D for 4 weeks caused some significant improvement in 2,4-D-induced antioxidant defense by increasing the antioxidant activity of the enzyme and reducing MDA and the CD levels (*p *< 0.05). Several studies have demonstrated the ability of olive oil to inhibit oxidative stress in the liver through various mechanisms [42,44]. Moreover, we have shown that the oral supplementation of olive oil to rats administered ethanol chronically restored damage caused to the liver by inhibiting lipid peroxidation and improving enzymatic activities [45]. The mechanism proposed to explain the positive effects of olive oil may be attributed to its richness in MUFA, mainly oleic acid which has different effects on lipid profiles and peroxidation in rabbit hepatic mitochondria [46]. However, the obtained data showed that EVOO and OOHF were more effective in alleviating 2,4-D- induced oxidative stress in the liver. In addition, concerning the OOLF group treated without 2,4-D, data showed that at variance with EVOO and OOHF, it increases serum γ-GT and liver MDA and decreases liver CAT and GR compared to controls. A possible explanation for these different effects is ascribed to oil's contents. Even though EVOO and OOLF fatty acid analysis revealed the same amount of MUFA but a higher content of unsaponifiable components such as polyphenols which may contribute to olive oil's beneficial effect. Thus, OOLF's toxic effects could be related to the lipid component administered without phenols. Indeed, EVOO contains a considerable amount of oleuropein, hydroxytyrosol, tyrosol and caffeic acid which all have potent inhibition effects against ROS [47,48]. Hydroxytyrosol is highly effective against DNA damage by peroxynitrite *in vitro *[21]. Caffeic acid phenethyl ester and its related compounds limit the functional alterations of the isolated mouse brain and liver mitochondria submitted to *in vitro *anoxia-reoxygenation [48].

Lipid peroxidation is the process of oxidative degradation of PUFA and its incidence in biological membranes resulting in impaired membrane function, structural integrity, decreased membrane fluidity and the inactivation of several membrane-bound enzymes [49]. Therefore, some particular attention was given to the liver's fatty acid composition in rats used in the current experiment. The highly increased levels of SFA and the decreased level of unsaturated fatty acids (UFA) characterized the liver's fatty acid composition of 2,4-D-treated rats compared to controls. As a consequence, a significant increase in the index of fatty acid unsaturation (SFA/UFA ratio), indicator of cell-membrane viscosity [50] was observed in the 2,4-D exposed groups. In rats, EVOO and its fractions intake led to significant changes in the hepatic fatty acid profile compared to controls and 2,4-D-treated animals. However, the type of fat in the diet accounted for changes in the rats' hepatic fatty acid composition. The standard diet is rich in corn oil and thus contains a high content of PUFA (mainly linoleic acid at 54%). That's why there was a decrease in the MUFA/PUFA ratio in the livers of controls, 2,4-D- and hydrophilic extract-treated rats. However, EVOO and its lipophilic extract which contain a high amount of MUFA (oleic acid 60%) increased the liver lipid MUFA content and decreased the PUFA level. As a consequence, a significant improvement was seen in the MUFA/PUFA ratio in the livers of rats fed EVOO and lipophilic extract treated or not with 2,4-D. In healthy humans, the short-term consumption of olive oil decreased serum oxidative stress [51] and their isolated lipoprotein fractions; LDL and HDL were shown to be enriched with oleic acid and resistant to oxidation [52,53]. Moreover, PUFAs are more susceptible to peroxidation resulting in MDA formation in mammalian tissues [54]. In fact, because of their peculiar structure - that is the presence of one or more double bonds-UFA are more susceptible to free radical damage and thus could increase the susceptibility of LDL particles to oxidation. Most of the studies comparing the effects of a MUFA-rich diet with PUFA-rich diet on LDL oxidation parameters have found a higher resistance of LDL particles to oxidation after the consumption of MUFA-rich diet [55,56]. Finally, the healthy effects of dietary MUFA, including lower endothelial activation [15] and susceptibility of LDL to oxidation [55,56] are indeed to be considered. Nevertheless, it is also remarkable to establish the amount and quality of phenolic compounds in extra virgin olive oil.

## Conclusion

The results of the present study showed that extra virgin olive oil and its extracts protect against oxidative damage of hepatic tissue by preventing excessive lipid peroxidation to increase MUFA composition and by maintaining serum marker enzymes and hepatic antioxidant enzyme activities at near normal concentrations. It is the hydrophilic fraction of olive oil which seems to be the effective one in reducing 2,4-D- induced oxidative stress, indicating that hydrophilic extract may exert a direct antioxidant effect on hepatic cells.

## List of Abbreviations

EVOO: extra virgin olive oil; OOLF: olive oil lipophilic fraction; OOHF: olive oil hydrophilic fraction; 2,4-D: 2,4-Dichlorophenoxiacetic acid; SOD: superoxide dismutase; GPx: glutathione peroxidase; GR: glutathione reductase; CAT: catalase; CD: conjugated dienes; MDA: malondialdehyde; SFA: saturated fatty acid; MUFA: monounsaturated fatty acid; PUFA: polyunsaturated fatty acid; UFA: unsaturated fatty acid; HDL: high density lipoprotein; LDL: low density lipoprotein.

## Competing interests

The authors declare that they have no competing interests.

## Authors' contributions

AN carried out the studies, acquired the data, performed the data analysis, drafted and revised the manuscript. WT and AG played a major role in all the experimental procedures of this study. MI carried out olive oil analysis. SD carried out olive oil analysis and provided technical assistance in the preparation of the manuscript and revised it. IC was involved in the experimental work performed towards this manuscript. ME provided the EVOO. AM carried out biochemical analysis. MH involved in the design and organization of the study, interpreted the results and revised the manuscript. All authors have read and approved the final manuscript.
